# Deadly Alliances: Death, Disease, and the Global Politics of Public Health

**DOI:** 10.1371/journal.pmed.0020004

**Published:** 2005-01-25

**Authors:** Matthew Gandy

## Abstract

Most people threatened by AIDS, tuberculosis, and unsafe drinking water are poor and have little or no influence over the global politics of public health

The rancour surrounding the Bangkok AIDS summit of July 2004 has exposed a series of fundamental disagreements surrounding the global politics of public health. These tensions range from access to cheaper lifesaving drugs to disputes over the role of poverty and gender equality in the promotion of sexual health.

The world is now experiencing the most profound public health challenge of the last forty years: we have witnessed the appearance of new diseases such as Ebola, SARS, and in particular, AIDS, combined with the alarming resurgence of diseases previously thought to have been under control, such as malaria and tuberculosis [1,2]. The AIDS pandemic in particular threatens to devastate entire regions and has already fundamentally altered the life expectancy and demographic profile of many countries in sub-Saharan Africa. Billions of people lack access to adequate sanitation and safe drinking water (Figure 1), and the UN predicts that slums will become the dominant urban form within the next fifteen years (Figure 2) [3,4,5]. 

**Figure 1:**
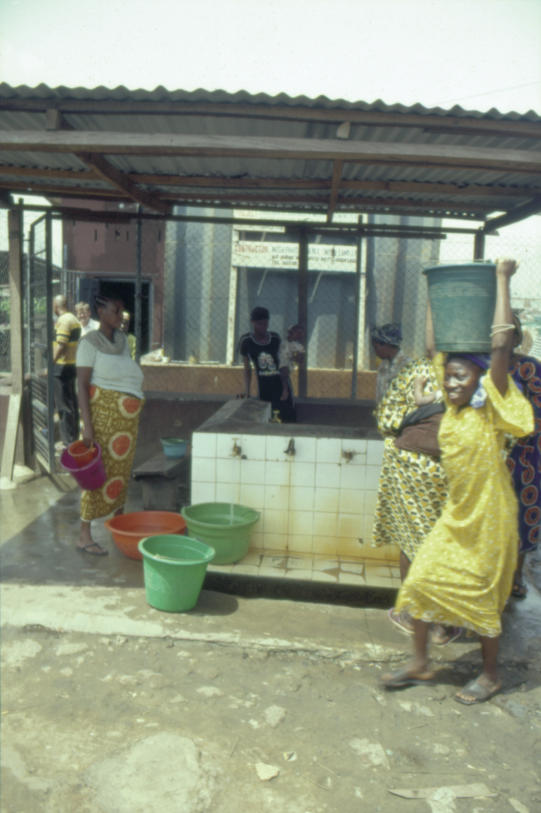
A Recently Completed Water Supply Project in Amukoko, Lagos, Nigeria Unsafe drinking water is the major cause of disease in Lagos. Under 5% of households receive a piped water supply and under 1% are connected to a closed sewer system. (Photo: Matthew Gandy)

**Figure 2:**
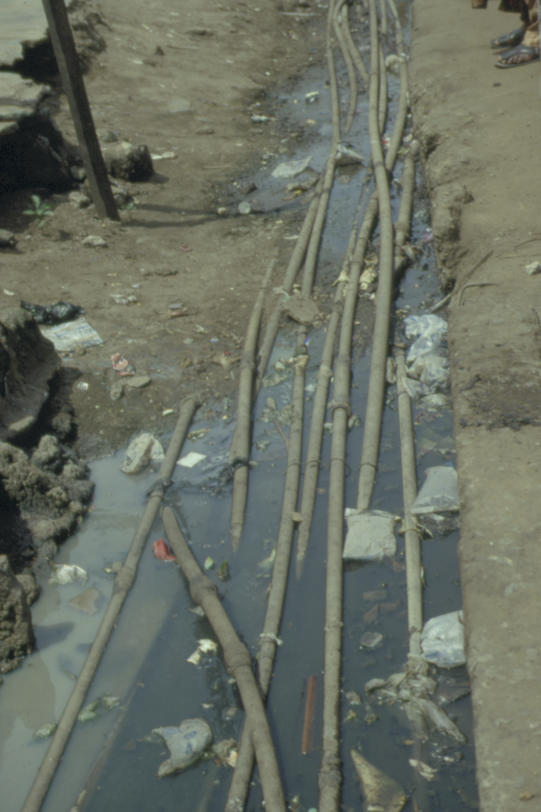
Open Sewer with Drinking Water Pipes Passing Through This photograph was taken in Amukoko, one of the largest slum areas in Lagos, Nigeria, a rapidly growing megacity with an estimated population of 15 million. Lagos is predicted to be one of the world's largest cities within the next 10 years. (Photo: Matthew Gandy)

Yet the very idea of “public health” sits uncomfortably alongside the current emphasis of bio-medical science on the molecular realm of DNA coding and the development of lucrative Western markets for new pharmaceutical products such as sildenafil (Viagra). The needs of the majority—the global poor—scarcely feature within this tactical alliance between the biomedical sciences and corporate power. Since most people threatened by AIDS, tuberculosis, unsafe drinking water, and other health threats are poor, they have little or no influence over the global politics of public health.

## Making Sense of the Crisis

If we are to make sense of the current public health crisis, we need to explore interconnections between political, economic, and social developments that are ignored by the fragmentary emphasis of the biomedical sciences. The current impetus toward economic globalization is causing widespread social and economic disruption, ranging from wild currency fluctuations to the systematic collapse of viable agricultural systems [6]. The imposition of austerity packages—widely referred to as structural adjustment programmes—in combination with the forcible extension of global markets for Western products is plunging millions of people into poverty and economic dependence. Existing primary health care services in much of the developing world and the former states of the Soviet Union have been drastically cut back, and services that were once freely available are now increasingly beyond the reach of the poor. And these harmful trends also extend even to the wealthiest global cities, such as London and New York, where a combination of poverty, homelessness, and cutbacks in primary health care since the 1980s has contributed toward the spread of tuberculosis and other diseases [7,8].

An examination of the social impact of the global public health crisis shows that it is women and children who have been most badly affected. One of the least addressed dimensions to the externally imposed structural adjustment programmes on developing countries is the harmful impact on the sexual health of women caused by their increased economic dependence on men. Sexual health programmes based on abstinence, for example, ignore the difficulties women face in negotiating safer or non-penetrative forms of sex. It is married women in southern Africa and south Asia who make up the largest and most vulnerable group of women, since they are at risk of being infected by their husbands.

A new generation of HIV-positive women activists, such as the Nigerian journalist and AIDS campaigner Rolake Odetoyinbo Nwagwu, are now engaged in a vital struggle to challenge the social attitudes and economic inequalities that have driven the devastating impact of AIDS on women and children in developing countries. The spread of AIDS in more traditional societies is closely linked with patriarchal power structures.. The sustenance of these structures is assured by the rise of poverty-fuelled ethnic and religious chauvinism, which undermines the prospects for developing more progressive approaches to social policy. And in regions where war or civil strife prevail, the vulnerability of women and children to sexual violence, economic exploitation, and disease is even greater so that we cannot consider public health questions separately from issues surrounding political stability and social justice.

## Spaces of Exclusion

In order to understand better the political dynamics behind public health we need to recognize that the development of modern forms of governance has emerged in tandem with new approaches toward the administration of human populations. The rise of the 19th-century industrial city, for example, necessitated the development of much more sophisticated forms of urban governance in order to tackle the threat of epidemic disease and enable these new cities to function effectively as centres of economic activity. But these new spaces of public health control, which scholars such as Michel Foucault have so vividly described, had a shadowy “other”. This “other” was represented by the increasingly squalid conditions endured by the mass of the population in European colonies, exemplified by the devastating modern outbreaks of bubonic plague in cities such as Baghdad, Lagos, and Bombay [9].

The Italian political philosopher Giorgio Agamben has elaborated on this distinction between “inside” and “outside” under modern systems of governance to reveal the persistence of what he terms conditions of “bare life” within even the most sophisticated legal and political systems [10]. The contemporary exclusion of the world's poor from adequate medical care is thus a form of state-sponsored violence, in which millions are denied even the most basic human rights. These “wasted lives”, to use the sociologist Zygmunt Bauman's phrase, represent a literal as well as metaphorical process of permanent and deadly exclusion for the poor, the marginalized, and others who have no value within the global economy [11].

## Excluding the Poor from Medical Care

A striking manifestation of this systematic exclusion of the poor from medical care is provided by the Bush administration's efforts to stymie access to affordable antiretroviral drugs. A dramatic standoff in 2001 between the global pharmaceutical industry and public health activists in South Africa, following the import of cheap generic antiretroviral drugs manufactured in Brazil, led to the historic Doha Declaration on intellectual property rights and public health [12]. Generic drug production in countries such as India, Brazil, and Thailand has succeeded in bringing down treatment costs per patient from $10,000 to $300 per year, yet the World Health Organization has revealed that less than one in 20 people who need antiretroviral treatment in the developing world are currently receiving it [13].

In order to widen access to affordable drugs, the current production of generic medicines will have to be massively expanded, but the Bush administration and its corporate allies in the pharmaceutical industry has worked assiduously to undermine the potential impact of the Doha agreement [14]. The United States government, for example, has negotiated bilateral trade deals with countries such as Chile and Thailand in an effort to dissuade them from the production of cheaper drugs [15]. Tensions exploded into the open at the Bangkok AIDS summit of 2004, where lobbyists on behalf of the US government put forward a series of specious assertions about the distribution of much-needed AIDS drugs in sub-Saharan Africa. The lobbyists asserted that the costs of drugs have been lowered—yet discounted brands remain at least twice as expensive as generic drugs. And the lobbyists for the Bush administration claimed that generic drugs undermine profitability and hence the incentive for new research (which is, in any case, overwhelmingly focused on the bloated market for prescription drugs in the US).

Of the $15 billion pledged by the Bush administration in the fight against AIDS most of this money will be focused on 15 selected countries. The countries were chosen by their willingness to abide by bilateral trade deals to prevent the production of cheaper generic drugs and their willingness to stress the centrality of abstinence as a strategy for AIDS prevention (a strategy that the religious Right has pushed for) [16,17]. It should also be noted that some of the most vocal critics of US policy, among them France, currently make a derisory financial contribution to global efforts to tackle HIV/AIDS so that the inadequacies of US policy must be viewed in a wider context of Western negligence toward the public health needs of the world's poorest countries [18].

The US government has sought to highlight the inadequacies of health care infrastructure in developing countries as a further justification for the diversion of attention from the cost of drugs. But primary health care services have themselves been undermined by the structural adjustment and trade policies promoted by Western financial institutions and their corporate backers. The global politics of AIDS is therefore caught in a neo-liberal vicious spiral from which it is impossible to disentangle the needs for social and institutional reform in the worst affected regions from the challenge of widening access to available treatments.

## The Contemporary Politics of Public Health

Public health pioneers of the past, such as the German bacteriologist Robert Koch, were not only scientists but also political advocates for social change. Improvements in health care were perceived as part of a nexus of reforms that ranged from better housing and nutrition to an extension of voting rights to ensure that the poor had adequate political representation.

The contemporary politics of public health needs to be considered, however, in relation to wider discourses on security and human welfare that are quite different from those of the 19th century. The current Western preoccupation with the threat of international terrorism, for example, has distracted attention from the much more real threats to human well-being created by poverty and ill health. The vast resources—both financial and logistical—now being poured into “security” contrast starkly with the inadequate funding provided for the Global Fund for HIV/AIDS, Tuberculosis, and Malaria. The increasingly unilateral and self-interested stance of the US illustrates the tensions between attempts to build international forms of governance for health care and the continuing centrality of national interests or global institutions that explicitly represent the economic power of a relatively small group of nations [19].

The paradox is that widening global disparities in wealth and poverty play a role in fostering the social and political conditions in which religious fanaticism and hatred for the West can flourish. So any “war on terror” that fails to address the causes of poverty, despair, and insecurity in the lives of the world's poor will ultimately create a more dangerous world for everyone.
